# Supplementary material for: National U.S. time-trends in opioid use disorder hospitalizations and associated healthcare utilization and mortality
Source: PLoS One. 2020 Feb 18;15(2):e0229174. doi: 10.1371/journal.pone.0229174 (PMC7028263; doi:10.1371/journal.pone.0229174)
Supplement: S1 Table — (DOCX) [file pone.0229174.s001.docx]

**S1 Table. OUD-hospitalization outcomes by age, sex and race**

|  | Total hospital charges  Mean (SE); median | Length of hospital stay  Mean (SE); median | % Discharged to non-home settings,  N (%) | Total hospital charges  >median,  N (%) | Length of hospital stay >3 days,  N (%) | Died during hospitalization,  N (%) |
| --- | --- | --- | --- | --- | --- | --- |
|  |  |  |  |  |  |  |
| **Age category, in years** |  |  |  |  |  |  |
| <34 | 19,391 (313); 9,280 | 3.2 (0.04); 1.5 | 43,787 (21.05%) | 73,411 (29.59%) | 64,217 (25.89%) | 5,963 (2.41%) |
| 34 - 45 | 19,958 (499); 9,831 | 3.4 (0.04); 1.7 | 33,663 (22.38%) | 63,307 (35.63%) | 51,860 (29.18%) | 4,284 (2.42%) |
| >45 - 55 | 26,850 (411); 14,012 | 3.9 (0.04); 2.0 | 34.309 (23.05%) | 76,215 (45.61%) | 58,458 (34.98%) | 3,903 (2.34%) |
| >55 | 31,146 (298); 18,449 | 4.2 (0.03); 2.5 | 50,051 (28.33%) | 99,568 (52.78%) | 78,813 (41.78%) | 4,229 (2.24%) |
| **Sex** |  |  |  |  |  |  |
| Male | 23,908 (370); 11,716 | 3.6 (0.03); 1.8 | 73,108 (21.10%) | 159,035 (39.00%) | 127,738 (31.33%) | 10,871 (2.67%) |
| Female | 23,859 (300); 12,737 | 3.7 (0.03); 2.0 | 88,638 (26.29%) | 153,372 (41.08%) | 125,518 (33.62%) | 7,523 (2.02%) |
| **Race** |  |  |  |  |  |  |
| White | 25,406 (238); 13,339 | 3.7 (0.02); 1.9 | 116,236 (25.32%) | 215,485 (41.40%) | 173,431 (33.32%) | 12,770 (2.46%) |
| Black | 24,873 (661); 12,397 | 3.9 (0.06); 2.0 | 10,004 (17.57%) | 26,722 (40.57%) | 22,691 (34.45%) | 1,349 (2.05%) |
| Hispanic | 28,781 (953); 14,214 | 3.8 (0.08); 1.8 | 7,759 (19.12%) | 22,602 (46.93%) | 15,801 (32.82%) | 1,154 (2.40%) |
| Other/missing | 16,469 (863); 8,220 | 3.3 (0.06); 1.7 | 27,822 (17.19%) | 47,759 (32.46%) | 41,489 (28.20%) | 3,116 (2.12%) |
