# Supplementary material for: National U.S. time-trends in opioid use disorder hospitalizations and associated healthcare utilization and mortality
Source: PLoS One. 2020 Feb 18;15(2):e0229174. doi: 10.1371/journal.pone.0229174 (PMC7028263; doi:10.1371/journal.pone.0229174)
Supplement: Attachment — Submitted filename: Response_Reviewers_R2_OUD.docx [file pone.0229174.s002.docx]

We thank the editors for their comments. Here are our responses to each comment.

Thank you for submitting your manuscript entitled "National U.S. Time-trends in Opioid Use Disorder Hospitalizations and Associated Healthcare Utilization and Mortality" to PLOS ONE.  Your manuscript files have been checked in-house but before we can proceed we need you to address the following issues:

1. We note that you have indicated that data from this study are available upon request. PLOS only allows data to be available upon request if there are legal or ethical restrictions on sharing data publicly. For information on unacceptable data access restrictions, please see <http://journals.plos.org/plosone/s/data-availability#loc-unacceptable-data-access-restrictions>.

* In your revised cover letter, please address the following prompts:

a) If there are ethical or legal restrictions on sharing a de-identified data set, please explain them in detail (e.g., data contain potentially identifying or sensitive patient information) and who has imposed them (e.g., an ethics committee). Please also provide contact information for a data access committee, ethics committee, or other institutional body to which data requests may be sent.

b) If there are no restrictions, please upload the minimal anonymized data set necessary to replicate your study findings as either Supporting Information files or to a stable, public repository and provide us with the relevant URLs, DOIs, or accession numbers. Please see <http://www.bmj.com/content/340/bmj.c181.long> for guidelines on how to de-identify and prepare clinical data for publication. For a list of acceptable repositories, please see <http://journals.plos.org/plosone/s/data-availability#loc-recommended-repositories>.

We will update your Data Availability statement on your behalf to reflect the information you provide.

Response: These data are owned by the U.S. Healthcare Cost and Utilization Project, are easily available from HCUP Nationwide Inpatient Sample (NIS) to any researcher interested in analyzing them. We do not own these data legally. According to data user agreement that we signed to obtain the data, we are not allowed to re-distribute or re-release these data to anyone (<https://www.hcup-us.ahrq.gov/db/nation/neds/NEDS_Introduction_2012.jsp#summary>). In our experience, obtaining the data from AHRQ was very easy and quick. We have added a data availability statement for interested readers who would like to obtain these data, along with the address available on the HCUP website.

# “Data Availability

These data are easily available from the "Healthcare Cost and Utilization Project (HCUP)" and can be obtained after completing an on-line Data Use Agreement training session and signing a Data Use Agreement. The contact information for requesting the data is as follows:

HCUP Central Distributor 
Phone: (866) 556-4287 (toll-free) 
Fax: (866) 792-5313 
E-mail: [HCUPDistributor@ahrq.gov](mailto:HCUPDistributor@ahrq.gov)”

2. Thank you for stating the following in the Competing Interests section:

"I have read the journal's policy and the authors of this manuscript have the following competing interests. JAS has received consultant fees from Crealta/Horizon, Medisys, Fidia, UBM LLC, Medscape, WebMD, Clinical Care options, Clearview healthcare partners, Putnam associates, Spherix, the National Institutes of Health and the American College of Rheumatology.  JAS owns stock options in Amarin pharmaceuticals and Viking therapeutics. JAS is a member of the executive of OMERACT, an organization that develops outcome measures in rheumatology and receives arms-length funding from 36 companies. JAS serves on the FDA Arthritis Advisory Committee. JAS is a member of the Veterans Affairs Rheumatology Field Advisory Committee. JAS is the editor and the Director of the UAB Cochrane Musculoskeletal Group Satellite Center on Network Meta-analysis.  JAS previously served as a member of the following committees: member, the American College of Rheumatology's (ACR) Annual Meeting
Planning Committee (AMPC) and Quality of Care Committees, the Chair of the ACR Meet-the-Professor, Workshop and Study Group Subcommittee and the co-chair of the ACR Criteria and Response Criteria subcommittee.  JDC has no conflicts. ".

i) Please confirm that this does not alter your adherence to all PLOS ONE policies on sharing data and materials, by including the following statement: "This does not alter our adherence to  PLOS ONE policies on sharing data and materials.” (as detailed online in our guide for authors <http://journals.plos.org/plosone/s/competing-interests>).  If there are restrictions on sharing of data and/or materials, please state these. Please note that we cannot proceed with consideration of your article until this information has been declared.

ii) Please include your updated Competing Interests statement in your cover letter; we will change the online submission form on your behalf.

Please know it is PLOS ONE policy for corresponding authors to declare, on behalf of all authors, all potential competing interests for the purposes of transparency. PLOS defines a competing interest as anything that interferes with, or could reasonably be perceived as interfering with, the full and objective presentation, peer review, editorial decision-making, or publication of research or non-research articles submitted to one of the journals. Competing interests can be financial or non-financial, professional, or personal. Competing interests can arise in relationship to an organization or another person. Please follow this link to our website for more details on competing interests: <http://journals.plos.org/plosone/s/competing-interests>

Response: We have added this to competing interest statement. The revised competing interest statement is as follows and is included in the cover letter:

“**Competing Interests**:

I have read the journal's policy and the authors of this manuscript have the following competing interests. JAS has received consultant fees from Crealta/Horizon, Medisys, Fidia, UBM LLC, Trio health, Medscape, WebMD, Clinical Care options, Clearview healthcare partners, Putnam associates, Spherix, Practice Point communications, the National Institutes of Health and the American College of Rheumatology. JAS owns stock options in Amarin pharmaceuticals and Viking therapeutics. JAS is on the speaker’s bureau of Simply Speaking. JAS is a member of the executive of OMERACT, an organization that develops outcome measures in rheumatology and receives arms-length funding from 12 companies. JAS serves on the FDA Arthritis Advisory Committee. JAS is a member of the Veterans Affairs Rheumatology Field Advisory Committee. JAS is the editor and the Director of the UAB Cochrane Musculoskeletal Group Satellite Center on Network Meta-analysis. JAS previously served as a member of the following committees: member, the American College of Rheumatology's (ACR) Annual Meeting Planning Committee (AMPC) and Quality of Care Committees, the Chair of the ACR Meet-the-Professor, Workshop and Study Group Subcommittee and the co-chair of the ACR Criteria and Response Criteria subcommittee.  JDC has no conflicts.

**This does not alter our adherence to PLOS ONE policies on sharing data and materials**.”

3. Your ethics statement must appear in the Methods section of your manuscript. If your ethics statement is written in any section besides the Methods, please move it to the Methods section and delete it from any other section. Please also ensure that your ethics statement is included in your manuscript, as the ethics section of your online submission will not be published alongside your manuscript.

Response: We have added this to the methods section, as recommended.

“The University of Alabama at Birmingham’s Institutional Review Board approved this study (X120207004) and waived the need for informed consent for this database study since these national data are de-identified. All investigations were conducted in conformity with ethical principles of research.”
